# Modulation of Poultry Cecal Microbiota by a Phytogenic Blend and High Concentrations of Casein in a Validated In Vitro Cecal Chicken Alimentary Tract Model

**DOI:** 10.3390/vetsci11080377

**Published:** 2024-08-16

**Authors:** Igor V. Popov, Nouhaila Belkassem, Ruud Schrijver, Iuliia P. Chebotareva, Michael L. Chikindas, Alexey M. Ermakov, Koen Venema

**Affiliations:** 1 Centre for Healthy Eating & Food Innovation (HEFI), Maastricht University—Campus Venlo, 5928 SZ Venlo, The Netherlands; nouhaila.belkassem@gmail.com (N.B.); koen.venema@wur.nl (K.V.); 2 Faculty “Bioengineering and Veterinary Medicine” and Center for Agrobiotechnology, Don State Technical University, 344000 Rostov-on-Don, Russia; 3 Animal Health Concepts BV, 8141 GN Heino, The Netherlands; 4 Health Promoting Naturals Laboratory, School of Environmental and Biological Sciences, Rutgers State University, New Brunswick, NJ 08901, USA; 5 Department of General Hygiene, I.M. Sechenov First Moscow State Medical University, 119435 Moscow, Russia

**Keywords:** CALIMERO-2, in vitro cecal model, phytogenic blends, 16S rRNA, poultry

## Abstract

**Simple Summary:**

Plant-derived compounds, also known as phytogenics, are gaining popularity in the poultry industry as an effective replacement for antibiotic growth promoters, whose wide and irrational use damages the ecosystem and health of animals and humans. Phytogenics are known for improving poultry’s health and production performance. These effects are closely related to the cecal microbiota of chickens, which highlights the importance of studying the effects of any feed additive related to gut microbial communities. In this study, we assessed the effects of a phytogenic blend with and without casein in high amounts on cecal microbiota composition and diversity using an artificial gastrointestinal system (CALIMERO-2) that mimics the physiology of a chicken’s cecum. The phytogenic blend promoted the abundance of bacteria associated with energy metabolism and production performance in poultry and decreased the presence of opportunistic pathogens. This study showed promising feed additives that can be used as growth promoters for poultry; however, testing in living broiler chickens to prove these data is needed, as artificial GI systems cannot fully reproduce the intestinal physiology of animals.

**Abstract:**

Phytogenic blends (PBs) consist of various bioactive plant-derived compounds that are used as growth promoters for farm animals. Feed additives based on PBs have beneficial effects on farm animals’ production performance, health, and overall well-being, as well as positive modulating effects on gut microbiota. In this study, we used a validated in vitro cecal chicken alimentary tract model (CALIMERO-2) to evaluate the effects of a PB (a mix of components found in rosemary, cinnamon, curcuma, oregano oil, and red pepper), alone or in combination with casein (control), on poultry cecal microbiota. Supplementation with the PB significantly increased the abundance of bacteria associated with energy metabolism (*Monoglobus*) and growth performance in poultry (*Lachnospiraceae* UCG-010). The PB also decreased the abundance of opportunistic pathogens (*Escherichia-Shigella*) and, most importantly, did not promote other opportunistic pathogens, which indicates the safety of this blend for poultry. In conclusion, the results of this study show promising perspectives on using PBs as feed additives for poultry, although further in vivo studies need to prove these data.

## 1. Introduction

Phytogenic blends (PBs), otherwise known as phytogenics, phytobiotics, or phytochemicals, consist of various bioactive plant-derived compounds and are used in livestock as feed additives. There are more than 5,000 individual dietary phytogenics that have been discovered in vegetables, fruits, herbs, legumes, whole grains, nuts, and essential oils [1]. Feed additives based on PB have beneficial effects on animals’ health, well-being, and overall production performance due to their anti-inflammatory, antioxidant, and antimicrobial properties [2,3]. Given their beneficial modes of action on livestock animals, PBs are recognized as growth enhancers and natural alternatives to antibiotic growth promoters [4]. It also should be noted that in contrast to antibiotic growth promoters, feed additives based on PBs do not lead to resistance development, have no withdrawal time in view of residues in animal products, and have minimal overall side effects, which results in the improvement of the quality of products derived from livestock animals and also contributes to environmentally sustainable and eco-friendly agriculture [5,6,7].

The beneficial effects of PBs on animals’ health are connected to the modulatory effects on gut microbial communities, which are able to metabolize the phytogenic compounds and, in turn, affect the hosts’ physiology through changes in microbial composition, diversity, and metabolite production [1,8]. Numerous studies have shown that the gut microbiome plays a key role in farm animals’ production performance as it directly influences the immune system, intestinal morphology, and gastrointestinal physiology, which marks the importance of testing the effects of any feed additives on intestinal microbiota [9,10,11,12].

In this study, we assessed the effects of a PB, casein (as a positive control), and their combination on poultry gut microbiota in vitro. It is generally accepted that the most important microbial fermentation processes in chickens occur in the cecum [13,14,15]. The targeted modulation of the cecal microbiome in poultry is significantly associated with an improvement in the growth performance and overall health of the animals [9,10,13]. This is why feed additives designed to modulate the poultry microbiome should target the microbial communities of this part of the GIT. To test the above supplements, we used the in vitro validated cecal chicken alimentary tract model (CALIMERO-2) based on TIM-2, which is used to mimic cecal physiology and microbial communities and to test feed additives and poultry nutritional products [16].

## 2. Materials and Methods

### 2.1. Study Products

A phytogenic blend (PB, Animal Health Concepts, Heino, the Netherlands) and casein were added to the standard ileal efflux medium (SIEM) for the CALIMERO-2 experiments (Table 1). Supplementation only with SIEM was used as a control and consisted of starch, arabinogalactan, pectin, amylopectin, xylan, vitamins, protein (casein), salts, ox bile, and Tween 80, as described by Cuevas-Tena et al. [17]. The PB contained a mix of herbal extracts and essential oils, including components found in cinnamon, rosemary, red pepper, turmeric (curcuma), and oregano oil.

### 2.2. Cecal Content Samples Collection and Preparation

Cecal content was obtained from several broiler chickens (Ross 308) from slaughterhouse van der Linden Poultry Products B.V. (Beringe, the Netherlands) as described before [16]. To prepare a standardized inoculum of microbiota, samples of cecal content were homogenized in an anaerobic cabinet (Sheldon Lab–Bactron IV, Gomelius, OR, USA) [18]. The obtained cecal content slurries were then snap-frozen in 35 mL portions with liquid nitrogen and then stored at −80 °C. Prior to inoculation, 4 tubes of 35 mL of cecal slurry were thawed for 1 h at 37 °C, added to the pre-reduced dialysate (the same dialysate used for the dialysis but with removed oxygen), and subsequently mixed to a 250 mL total volume.

### 2.3. Experimental Setup and the CALIMERO-2

The in vitro model has been extensively used over the past two decades and has been shown to be very reproducible [18]. In each CALIMERO-2 unit, 60 mL of pooled standardized microbiota and pre-reduced dialysate were inoculated. Then, SIEM was administered to each unit (2.5 mL/h) for an adaptation period of 16 h. After the adaptation period, all CALIMERO-2 units were supplemented with a constant SIEM flow (2.5 mL/h) for 72 h according to the conditions described in Table 1. The simulation of the passage from the cecum to the distal colon was simulated every 24 h by removing 25 mL of lumen content from CALIMERO-2 units. The analysis of microbiota composition (Section 2.4) was conducted using lumen samples collected at 0, 24, 48, and 72 h of the experiment. The number of replications for every study product was three. The experiments were performed as described by Oost et al. [16].

### 2.4. Gut Microbiota Composition

High-throughput V3–V4 16S rRNA sequencing was performed to assess microbiota composition and diversity, as described before [17]. In short, DNA was extracted from the CALIMERO-2 lumen samples, amplified, barcoded, pooled, and then sequenced using the Illumina MiSeq (Illumina, Eindhoven, the Netherlands) according to the manufacturer’s instructions. The bcl2fastq (v. 1.8.3, Illumina, San Diego, CA, USA) software was used to obtain FASTQ files based on the conversion of the sequences after quality checking. QIIME 2 (v. 2023.9) software was used to analyze the results [19]. The denoising and generation of amplicon sequence variants database were performed using DADA2 [20] implemented in QIIME 2. The taxonomical identification of amplicon sequence variants was performed with the reference 16S rRNA SILVA database (version 138 (available online: https://www.arb-silva.de/documentation/release-138; accessed on 23 January 2023)) [21]. Alpha diversity metrics (the Shannon’s index [22], Chao1 index [23], and Pielou’s evenness [24]), and beta diversity indexes (Bray–Curtis dissimilarity [25], and Jaccard similarity [26]) were also calculated.

### 2.5. Statistical Analysis

The statistical analysis of microbiota data was performed with the programming language R (v4.2.3, R Foundation for Statistical Computing, Vienna, Austria). Before the hypothesis testing, we performed filtering of the bacterial taxa prevalence by applying a minimal 10% threshold to obtain more robust results. Subsequently, relative abundances of bacterial taxa were calculated based on acquired counts data. To determine differences in the bacterial relative abundances and alpha diversity indexes, the Kruskal–Wallis test was used. Dunnet’s test was implemented for the multiple comparisons. Differences in beta diversity distances were analyzed with the PERMANOVA test implemented into the “adonis” function from the “vegan” (v2.6-6.1) package (the number of permutations was set to 1000). Beta diversity distances were visualized with principal coordinate analysis (PCoA) plots. Multiple comparisons were adjusted with the Benjamini–Hochberg false discovery rate. Adjusted *p*-values (*q*-values) were considered significant at *q* < 0.1. The results of the statistical analysis were visualized using the “ggplot2” (v3.5.1) package.

## 3. Results

### 3.1. In Vitro Cecal Microbiota Composition

**Figure 1:**
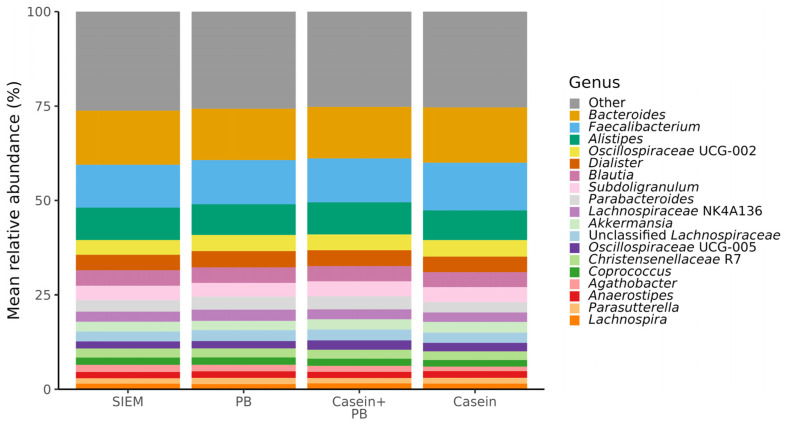
Composition (relative abundance) of identified taxa at the genus level of cecal microbiota from CALIMERO-2 units after interventions. SIEM = standard ileal efflux medium; PB = phytogenic blend.

Among the most dominant genera, whose relative abundance exceeded 1.5% in all CALIMERO-2 units, were *Bacteroides*, *Faecalibacterium*, *Alistipes*, *Oscillospiraceae* UCG-002, *Dialister*, *Blautia*, *Subdoligranulum*, *Parabacteroides*, *Lachnospiraceae* NK4A136, *Akkermansia*, unclassified *Lachnospiraceae*, *Oscillospiraceae* UCG-005, *Christensenellaceae* R7, *Coprococcus*, *Agathobacter*, *Anaerostipes*, *Parasutterella*, and *Lachnospira* (Figure 1).

**Figure 2:**
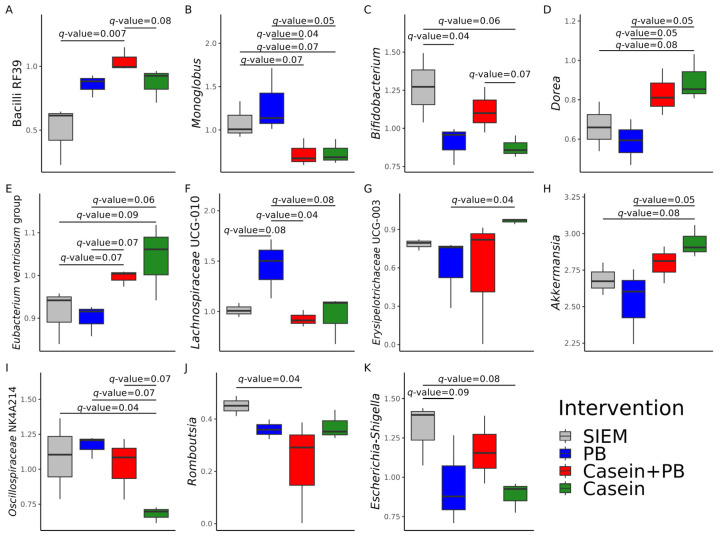
Filtered relative abundance of genera, whose relative abundances differ significantly between interventions: (**A**) Bacilli RF39, (**B**) *Monoglobus*, (**C**) *Bifidobacterium*, (**D**) *Dorea*, (**E**) *Eubacterium ventriosum* group, (**F**) *Lachnospiraceae* UCG-010, (**G**) *Erysipelotrichaceae* UCG-003, (**H**) *Akkermansia*, (**I**) *Oscillospiraceae* NK4A214, (**J**) *Romboutsia*, (**K**) *Escherichia*-*Shigella*. SIEM = standard ileal efflux medium; PB = phytogenic blend.

The relative abundance of 11 taxa significantly differed among the studied interventions (Figure 2). The relative abundance of Bacilli RF39 was higher in CALIMERO-2 units supplemented with casein + PB in comparison with SIEM (*q*-value = 0.007) and casein (*q*-value = 0.08) (Figure 2A). Supplementation with SIEM and PB resulted in a significantly higher growth of *Monoglobus* (*q*-value ≤ 0.07) relative to other interventions (Figure 2B). *Bifidobacterium* was significantly dominant in CALIMERO-2 units supplemented with SIEM (*q*-value ≤ 0.06) and casein + PB (*q*-value = 0.07) (Figure 2C). The relative abundances of *Dorea* and the *Eubacterium ventriosum* group were significantly higher (*q*-value < 0.1) after supplementation with casein and casein + PB solutions (Figure 2D,E). Supplementation with PB resulted in the highest relative abundance of *Lachnospiraceae* UCG-010 (Figure 2F). Casein significantly promoted (*q*-value = 0.04) the relative abundance of *Erysipelotrichaceae* UCG-003 in comparison with PB (Figure 2G) and the relative abundance of *Akkermansia* relative to PB (*q*-value = 0.05) and SIEM (*q*-value = 0.08) (Figure 2H). On the other hand, the relative abundance of *Oscillospiraceae* NK4A214 was among the lowest (*q*-value ≤ 0.07) in the CALIMERO-2 unit supplemented with casein (Figure 2I). There were significant differences in the relative abundance of *Romboutsia* (*q*-value = 0.04) between SIEM and casein + PB (Figure 2J). The relative abundance of *Escherichia-Shigella* was significantly higher in the control CALIMERO-2 unit in comparison with the units supplemented with PB (*q*-value = 0.09) and casein (*q*-value = 0.08) (Figure 2K).

### 3.2. In Vitro Cecal Microbiota Diversity

**Figure 3:**
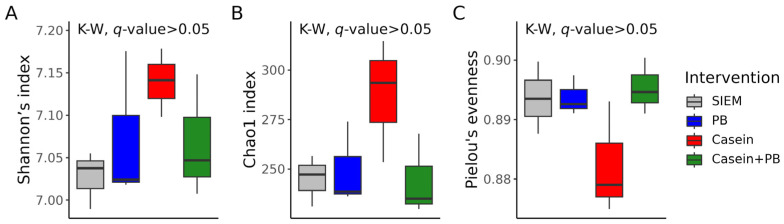
Alpha diversity of cecal microbiota from CALIMERO-2 units after interventions: Shannon’s index (**A**), Chao1 index (**B**), and Pielou’s evenness (**C**). The Kruskal–Wallis (K–W) test was used for the *q*-values calculation. SIEM = standard ileal efflux medium; PB = phytogenic blend.

According to the multiple comparisons testing of alpha diversity index values, there were no statistically significant differences between the interventions (Figure 3). Generally, the alpha diversity was more variable in CALIMERO-2 units supplemented with casein.

**Figure 4:**
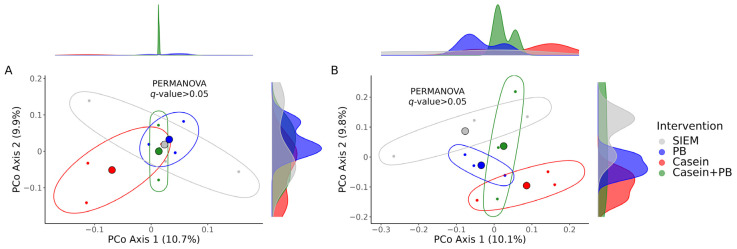
Principal coordinate analysis (PCoA) plots illustrating differences in the beta diversity: Bray–Curtis dissimilarity distances (**A**); Jaccard similarity distances (**B**). *q*-values were calculated with the PERMANOVA test; the number of permutations was set to 1000. The large colored spheres represent the centroids of the groups. SIEM = standard ileal efflux medium; PB = phytogenic blend.

We did not find any significant differences between the interventions concerning the beta diversity, although the distances between microbial communities in the CALIMERO-2 unit supplemented with the casein were notably prominent in comparison with other interventions according to the principal coordinate analysis (Figure 4).

## 4. Discussion

In this research, we studied the effects of the PB and its combination with casein on poultry cecal microbiota composition and diversity in an artificial, validated, dynamic, computer-controlled in vitro cecal system for (broiler) chickens (CALIMERO-2). CALIMERO-2 is an artificial GI model created based on the TNO in vitro model of the colon (TIM-2). It was validated in previous experiments with different feeding types; the microbial communities recreated in the CALIMERO-2 units were also compared with the original inoculum using high-throughput 16S rRNA amplicon sequencing. As a result, the bacterial compositions were reproducible and corresponded to the in vivo cecum, which makes this model a suitable platform for studying chickens’ cecum microbiota in response to in-feed interventions [16]. The application of such predictive bioreactor-based artificial GI systems in food and biotechnology studies allows for the medium- to high-throughput testing of products and their prompt optimization based on the acquired results [27]. The ability to conduct studies of formulations under development in an artificial gastrointestinal system with intestinal microbiota that closely mimics in vivo conditions avoids the use of multiple cohorts of animals during in vivo testing, which is not only cost-effective but also meets the modern requirements to reduce the involvement of laboratory animals in product testing [27,28]. Nevertheless, at the moment, in vitro modeling with artificial GI systems cannot fully replace in vivo studies, as these systems have obvious limitations, such as the inability to study host–gut microbiome interactions concerning the immune responses and overall effects of intestinal microbes on host physiology [29]. However, experiments with artificial GI systems provide valuable data that could allow us to choose the most promising product candidates and optimize them prior to the in vivo studies, contributing to the reduction of animal use in research [27,30].

Feed additives with PBs are used in the poultry industry as an alternative to antibiotic growth promoters as they effectively increase the production performance of chickens [31]. However, it should be mentioned that the effectiveness of each PB for poultry depends on their composition as well as the dosage of the supplements, the birds’ age, and the rearing environment [32]. Also, poultry production performance has a direct relationship with cecal microbiota, making microbial richness and composition one of the primary targets of feed supplements for poultry [33]. In our study, supplementation with a PB, casein, and their combination did not result in alterations in the alpha and beta diversity of poultry cecal microbiota in vitro, but these interventions affected the relative abundance of 11 taxa. The absence of effects on diversity indexes is probably related to the fact that all interventions did not affect the dominant taxa, whose overall relative abundance exceeded 1.5%, except for the *Akkermansia*. In our experiment, casein was used as a comparative control, as the beneficial effects of crude proteins, including casein, on chickens’ health, production performance, and gut microbiota have been previously investigated [34,35,36,37]. Also, casein is used as the protein source in SIEM preparation, and increasing its concentrations for comparative control experiments was the most suitable choice for the study’s reproducibility [16,30]. Given the fact that the supplementation of CALIMERO-2 units with casein did not significantly affect the relative abundance of the dominant taxa and the overall bacterial diversity, we speculate that the inoculated microbiome did not digest all supplementation to the extent that more prominent differences occurred. Nevertheless, the experiment was conducted within a validated model using the same pooled inoculated microbiota, which makes it possible to perform a direct comparison of microbiota-modulating effects of studied interventions and conclusions based on the acquired results.

The relative abundance of Bacilli RF39 was among the highest in the CALIMERO-2 unit supplemented with casein + PB solution. The comparative genomic study by Wang et al. showed that Bacilli RF39 can produce metabolites such as hydrogen and acetate but lacks some genes for important signaling pathways, for example for carbohydrate storage [38]. Supplementation with a PB resulted in the promotion of *Monoglobus* relative abundance in comparison with other experimental interventions. These bacteria showed the ability of pectin (polysaccharide) degradation, which is probably related to the source of PB and contributes to improving digestion and energy absorption in broilers [39,40]. The relative abundance of *Bifidobacterium* was lower in CALIMERO-2 units supplemented with PB and casein in comparison with the control, but the abundance of these bacteria did not have significant differences between the control and casein + PB. *Bifidobacterium* species are generally recognized as beneficial bacteria for poultry’s health and production performance [41,42]. *Dorea* was promoted by all interventions containing casein, which corresponds to previous studies, where diets with high amounts of protein also resulted in a higher relative abundance of *Dorea* in animal gut microbiota [43]. *Dorea* is a commensal bacteria in poultry’s cecal microbiota [44,45]. We have not found any studies of its functions in chicken microbiota, but in humans, *Dorea* is associated with obesity [46]. *Eubacterium ventriosum* group and *Erysipelotrichaceae* UCG-003, other taxa representing commensal cecal microbiota in poultry [47,48,49], were also promoted with casein-containing interventions. PB supplementation promoted *Lachnospiraceae* UCG-010, which was associated with greater growth performance in broiler chickens in previous studies [50]. Casein increased the relative abundance of *Akkermansia*. This taxon was previously shown to be associated with a decrease in body weight gain in humans [51,52]; however, according to the results of the Tolnai et al. study, this was not the case for poultry, as there were no significant associations between *Akkermansia* and broiler weight [53]. Casein supplementation also resulted in the lowest abundance of *Oscillospiraceae* NK4A214. *Oscillospiraceae* NK4A214 is the uncultured bacteria that was detected in the intestines of wild and farm animals, but its role in the gut microbiome is still unknown [54,55,56,57]. The relative abundance of *Romboutsia* was lower in the CALIMERO-2 unit supplemented with casein + PB solution in comparison with control. *Romboutsia* is usually identified in chicken’s jejunal and ileal microbiome, and recent studies have shown that species belonging to this genus have immunomodulatory properties [58,59]. Supplementation with PB and casein decreased the *Escherichia-Shigella* relative abundance. Bacteria belonging to these genera are gut commensals in humans and animals and sometimes happen to be pathogenic depending on specific strains [60,61].

## 5. Conclusions

In this study, we assessed the effects of the phytogenic blend (PB), with or without high amounts of casein as feed supplements, on poultry cecal microbiota using a validated in vitro cecal chicken alimentary tract model. There were no significant differences between the alpha and beta diversity indexes, which is related to the fact that all interventions did not affect the dominant taxa. However, there were prominent differences in the relative abundance of sub-dominant taxa. Remarkably, the phytogenic blend promoted the abundance of bacteria associated with energy metabolism and production performance in poultry (*Monoglobus* and *Lachnospiraceae* UCG-010) and decreased opportunistic pathogenic ones (*Escherichia-Shigella*). Overall, the results of this study show promising perspectives on using the studied phytogenic blend as a feed additive for poultry, although further in vivo studies need to prove these data.

## Figures and Tables

**Table 1:** Products supplemented to CALIMERO-2 units and their concentrations.

Run	Product	Concentration
1	SIEM	Standard [14]
2	SIEM + PB	500 μL PB *
3	SIEM + casein	12 g casein *
4	SIEM + PB + casein	12 g caseine + 500 μL PB *

* Added to SIEM per day.

## Data Availability

All raw sequence data associated with this study have been deposited in the National Center for Biotechnological Information’s Short Read Archive and are available under BioProject ID PRJNA1101453.
